# Comparing the impact on the prognosis of acute myocardial infarction critical patients of using midazolam, propofol, and dexmedetomidine for sedation

**DOI:** 10.1186/s12872-021-02385-9

**Published:** 2021-12-07

**Authors:** Xiaowei Jiang, Min Yan

**Affiliations:** 1 Cardiology Department, National Clinical Research Center for Geriatric Disorders, Xiangya Hospital Central South University, Changsha, Hunan China; 2 Internal Medicine Department, Changsha Medical University, Changsha, Hunan China

**Keywords:** Acute myocardial infarction, Sedative therapy, Mortality

## Abstract

**Background:**

There are less studies focusing on the sedative therapy of acute myocardial infarction (AMI) critical patients. This study aim to compare the impact on the prognosis of AMI critical patients of using midazolam, propofol and dexmedetomidine.

**Methods:**

We collected clinical data from the Medical Information Mart for Intensive Care III (MIMIC III) database. Data on 427 AMI patients with sedatives using were recruited from in Coronary Heart Disease Intensive Care unit (CCU).

**Results:**

There were 143 patients in midazolam using, 272 in propofol using and 28 in dexmedetomidine using. The rate of 28-days mortality was 23.9% in overall patients. Through logistic regression analysis, only midazolam using was significant association with increased 28-days mortality when compared with propofol or dexmedetomidine using. In the subgroup analysis of age, gender, body mass index (BMI), white blood cell (WBC), beta-block, and revascularization, the association between midazolam using and increased 28-days mortality remained significantly. Through propensity score matching, 140 patients using midazolam and 192 using non-midazolam were successfully matched, the midazolam using presented with higher rate of CCU mortality, hospital mortality and 28-days mortality, longer of mechanical ventilation time and CCU duration. E-value analysis suggested robustness to unmeasured confounding.

**Conclusion:**

Propofol or dexmedetomidine are preferred to be used in AMI critical patients for sedative therapy.

## Background

AMI critical patients’ primary concerns in CCU are respiratory and hemodynamic supports, and usually treated with many invasive therapies, which may cause discomfort and anxiety. Sedative therapy is assumed to reduce discomfort from care interventions, increase tolerance of mechanical ventilation, prevent accidental removal of instrumentation, and reduce metabolic demands during cardiovascular and respiratory instability [1]. Midazolam, propofol, and dexmedetomidine are widely used sedatives in clinical practice. Midazolam, a ganna-aninobutyric acid agonist, is a traditional sedative for critically ill patients and a short duration of effect. Dexmedetomidine is an alpha-2 adrenoreceptor agonist with a unique mechanism of action. Based on experimental myocardial infarction rats, midazolam was demonstrated with increased ventricular arrhythmias and death and infarct size following reperfusion [2], and dexmedetomidine with increased the cardiac infarct size [3], and propofol with myocardial protective effect by reducing release of inflammatory factors [4]. An small sample clinical study has demonstrated that sedation with dexmedetomidine and propofol may cause hypotension or bradycardia [5].

However, there are none clinical study focusing on the prognosis of different sedatives in AMI critical patients. The aim of our study is to compare the impact on the prognosis among midazolam, propofol and dexmedetomidine in AMI critical patients receiving sedative therapy.

## Methods

### MIMIC III database

Clinical information of patients in our study were collected from MIMIC III database, which was illustrated by the Massachusetts institute of technology and had over 40,000 patients admitted between 2001 and 2012 [6]. Patients in the database were fully anonymized. One author(X J) gained access involves MIMIC III database (certification number 9195641) and extracted the data.

### Inclusion criteria and exclusion criteria

Acute myocardial infarction patients diagnosed with AMI according to the 9th revision of the International Classification of Diseases Code (ICD-9) were initially screened. Patients with treatment records indicating sedatives using after CCU admission were initially screened. Sedatives included midazolam, propofol, and dexmedetomidine. Patients who were < 18 years or > 90 years old were excluded. For patients who had more than once CCU inpatient record, only the first CCU inpatients record was collected.

### Data extraction and missing data management

Data on the patients’ characteristics, past medical history, vital sign, biochemistry, sedatives and other treatments were recruited from the database. Variables with missing data are very common in the database of MIMIC III. Serum tropoin and RASS score, with more than 30% missing, were removed from this analysis. For continuous variables with less than 5% missing, we used imputation method with linear regression.

### Outcomes

The primary outcome was defined as 28-days mortality. The secondary outcomes included CCU mortality, hospital mortality, length of mechanical ventilation and CCU stay.

### Statistical analysis

Data analyses were performed using StataMP software version 16. Numeric variables were summarized as the mean (standard deviation). Categorical variables were reported as counts (percentage). The student’s test, ${\chi}^{2}$ test, Wilcoxon rank-sum test was used, as appropriate. Univariate and multivariate logistic regression were to explore significantly factors for 28-days mortality. The log-rank test was used to assess differences in 28-days mortality between groups divided by midazolam, propofol, and dexmedetomidine. Subgroup analysis was utilized with ${\chi}^{2}$ test to detect any interaction between midazolam and 28-days mortality, and stratification was performed according to age (< 60, ≥ 60), gender(male, female), BMI(< 24, ≥ 24), WBC(≤ 10, > 10), beta-block (Yes, No), and revascularization (Yes, No). Propensity score matching (PSM) could decrease the influence of confounding factors. The propensity score was allocated based on the probability of a patient who receive midazolam therapy and estimated with using a multivariable logistic regression model. The nearest neighbor matching algorithm was applied using a caliper width of 0.02. There were variables selected to establish the propensity score: age, male, hypertension, creatinine (Scr), myocardial infarction (NSTEMI, AWSTEMI, NAWSTEMI), beta-blocker, stain, vasopressor, and revascularization. Graph of the p score were used to examine the PSM degree. Finally, 140 patients from midazolam groups and 192 from non-midazolam groups were selected and used to further analyses. We explored the potential for unmeasured confounding by calculating E-values [6]. The E-value quantifies the required magnitude of an unmeasured confounder that could negate the observed association between midazolam and 28-days mortality. Two-sided P values less than 0.05 were considered statistically significant.

## Results

### Baseline clinical characteristics

Information about 427 AMI patients with sedative therapy were recruited. There were 143 patients with midazolam using, 272 patients with propofol using, 28 patients with the dexmedetomidine using, 53 patients with propofol and midazolam using, 22 patients with propofol and dexmedetomidine using, 13 patients with midazolam and dexmedetomidine using, and 8 patients with three seditives using. The overall 28-days mortality rate was 23.9%, and receiving mechanical ventilation was 93.4%. The comparisons of characteristics stratified by 28-days mortality are show in Table 1. There were no significant differences among the groups regarding sex, BMI, diabetes, PLT, using of clopidogrel and mechanical ventilation. Compared with 28-days survival, patients with 28-days mortality had older age, higher rate of hypertension and midazolam using and lower rate of using aspirin, betablocker, and revasucularization therapy(all *p* < 0.01).

**Table 1:** Comparisons of the clinical characteristics between groups stratified by 28-days mortality

Variables	N = 427	28-days		*p* value
		Mortality N = 102	Survival N = 325	
MI				0.179
NSTEMI	121(28.3)	32(31.4)	89(27.4)	
AWSTEMI	126(29.5)	35(34.3)	91(28.0)	
NAWSTEMI	180(42.1)	35(34.3)	145(44.6)	
Age, years	66.9 ± 12.3	69.4 ± 13.3	66.2 ± 11.9	0.020
Male, n(%)	304(71.8)	69(67.6)	238(73.2)	0.274
BMI, kg/m^2^	28.0 ± 5.5	28.1 ± 5.9	27.9 ± 5.4	0.871
Past medical history				
Hypertension, n(%)	55(12.9)	23(22.5)	32(9.8)	0.001
Diabetes, n(%)	138(32.3)	38(37.2)	100(30.8)	0.222
CHF, n(%)	49(11.5)	17(16.7)	32(9.8)	0.059
COPD, n(%)	77(1.6)	17(16.7)	60(18.5)	0.681
Peripheral, n(%)	70(1.6)	19(18.6)	51(15.7)	0.485
Heart rate, bpm	86.7 ± 14.5	89.6 ± 17.8	85.7 ± 13.2	0.019
MBP, mmHg	77.2 ± 8.1	75.3 ± 9.8	77.8 ± 7.4	0.007
SpO2, %	97.5 ± 2.2	96.7 ± 3.3	97.8 ± 1.6	0.000
*RASS score	0(− 2,0)	− 0.5(− 3,0)	0(− 1,0)	0.128
Biochemistry				
WBC, K/uL	14.1 ± 7.1	16.8 ± 8.7	13.3 ± 6.4	0.000
PLT, K/uL	220.7 ± 99.0	223.4 ± 101.5	219.8 ± 98.3	0.753
HGB, g/dl	10.8 ± 2.0	10.7 ± 1.9	10.8 ± 2.1	0.788
TB, mg/dl	1.1 ± 2.5	1.7 ± 4.8	0.9 ± 0.7	0.008
Glu, mg/dl	181.8 ± 105.1	229.9 ± 122.7	166.6 ± 94.2	0.000
Scr, mg/dl	1.3 ± 1.2	1.7 ± 1.2	1.2 ± 1.2	0.000
Potassium, mEq/L	4.3 ± 0.7	4.3 ± 0.7	4.3 ± 0.7	0.995
^&^Serum tropoin, ng/ml	4.3 ± 0.5	7.6 ± 1.3	2.9 ± 0.3	0.000
Treatments				
Aspirin, n(%)	323(75.6)	55(53.9)	268(82.5)	0.000
Clopidogrel, n(%)	142(33.3)	28(27.4)	114(35.1)	0.154
Betablock, n(%)	257(83.6)	58(56.9)	299(92.0)	0.000
Stain, n(%)	287(67.2)	41(40.2)	246(75.7)	0.000
Vasopressor, n(%)	335(78.5)	94(92.2)	241(74.2)	0.000
Mechanical ventilation, n(%)	399(93.4)	96(94.1)	303(93.2)	0.752
^#^Revascularization, n(%)	299(70.0)	47(46.1)	252(77.5)	0.000
Propofol, n(%)	272(63.7)	45(44.1)	227(69.8)	0.000
Midazolam, n(%)	143(33.5)	56(54.9)	87(26.8)	0.000
Dexmedetomidine, n(%)	28(6.5)	2(2.0)	26(8.0)	0.032
Propofol + Midazolam	53(12.4)	17(16.6)	36(11.1)	0.135
Propofol + Dexmedetomidine	22(5.1)	1(1.0)	21(6.5)	0.036
Midazolam + Dexmedetomidine	13(3.0)	1(1.0)	12(3.7)	0.318
All three seditives	8(1.9)	0(0)	8(2.5)	0.207

*MI* myocardial infarction; *NSTEMI* non-ST segment elevated myocardial infarction; *AWSTEMI* anterior wall ST segment elevated myocardial infarction; *NAWSTEMI* non-anterior wall ST segment elevated myocardial infarction; *BMI* body mass index; *CHF* congestive heart failure; *COPD* chronic obstructive pulmonary disease; *MBP* mean arterial pressure; *WBC* white blood cell; *PLT* platelet; *HGB* hemoglobin; *TB* total bilirubin; *Glu* glucose; *Scr* creatinine; *, 141 patients with RASS scores records, include 40 patients in mortality group and 101 in survival group; & 289 patients with serum tropoin record, include 201 patients in mortality group and 88 in survival group; #, include percutaneous coronary intervention and coronary artery bridge graft

### Worse prognosis in midazolam using groups

**Fig. 1:**
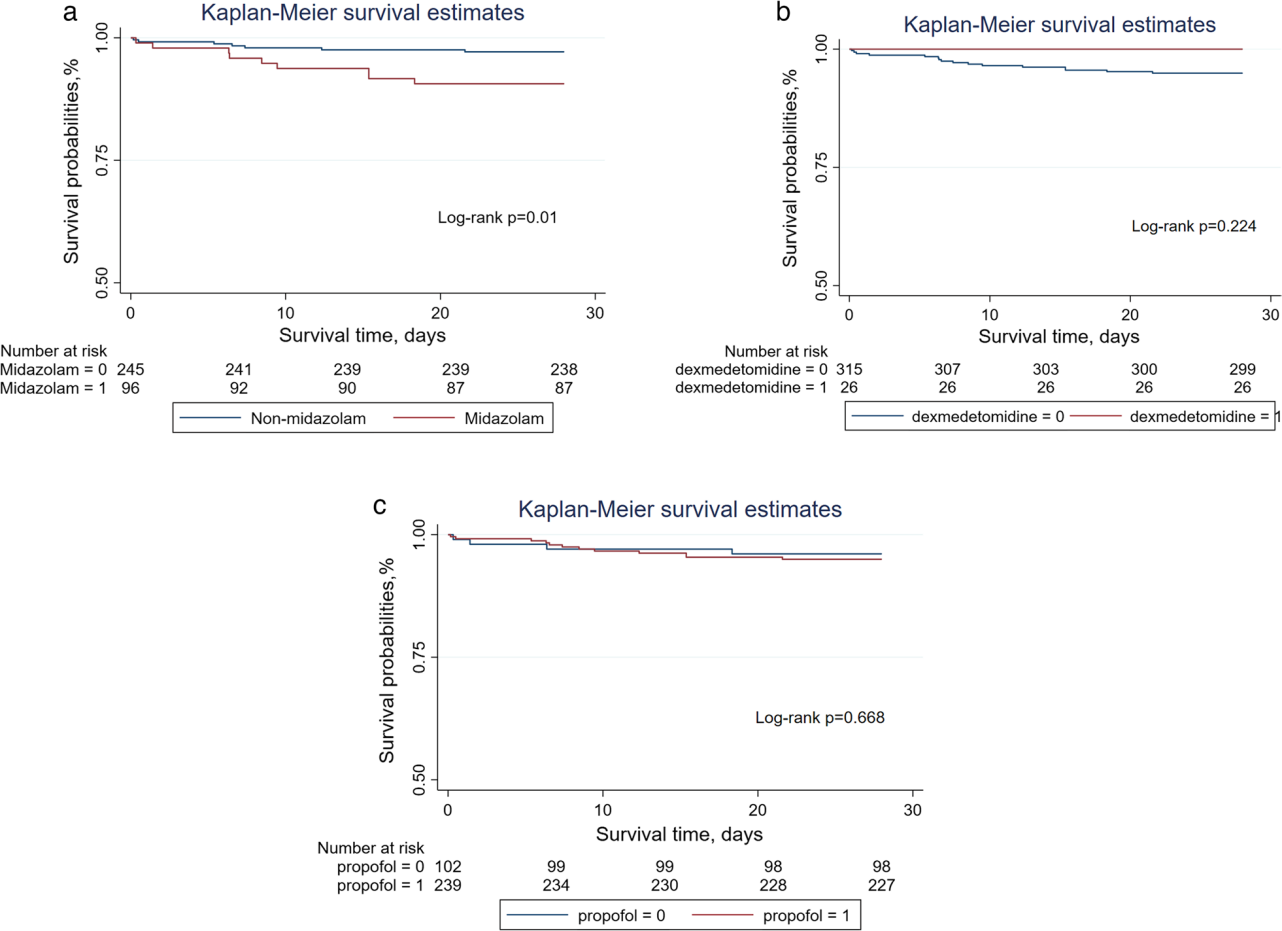
Kaplan–Meier method estimated 28-days mortality in patients with myocardial infarction stratified by midazolam (**A**), dexmedetomidine (**B**) or propofol (**C**). Patients with more than one seditive using were not included in Kaplan–Meier analysis

**Fig. 2:**
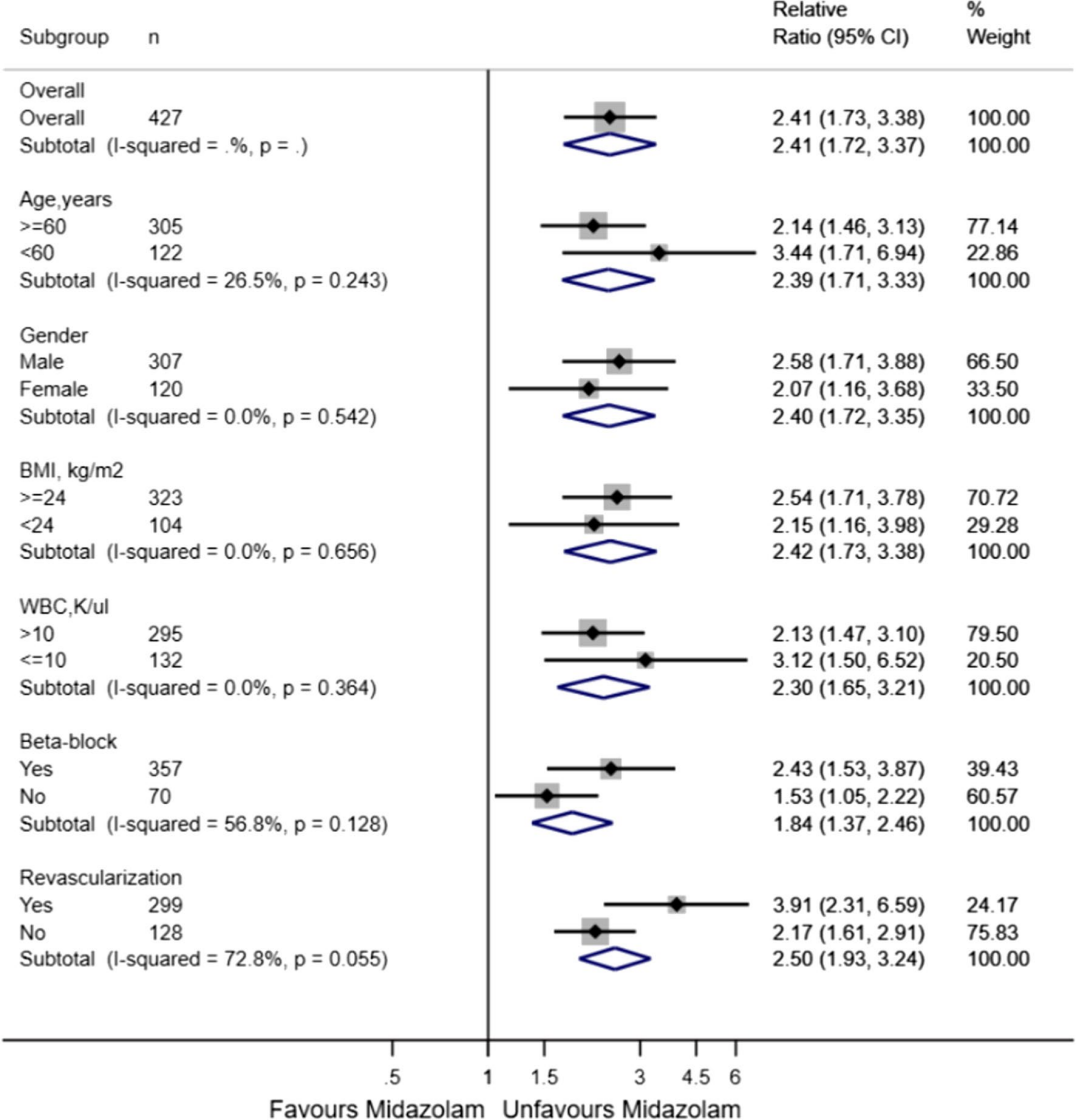
Subgroup analysis of the association between 28-days mortality and midazolam using

Through logistic regression analysis, we founded that only midazolam using was significant association with 28-days mortality among three sedatives (Table 2). The Kaplan–Meier curves revealed that a increased 28-days mortality was significantly associated with midazolam using (Fig. 1). In Table 3, when compared with non-midazolam using, the rate of CCU and hospital mortality were significantly higher, and the time of mechanical ventilation duration and CCU stay were significantly longer in midazolam using. Subgroup analysis was performed according to the age, gender, BMI, WBC, beta-block, and revascularization (Fig. 2). The HR of midazolam use was significant in the age subgroups(< 60 years old: HR 3.44, 95%CI 1.71–6.94; ≥ 60 years old: RR 2.14, 95%CI 1.46–3.13), gender subgroups(male: RR 2.58, 95%CI 1.71–3.88; female: RR 2.07, 95%CI 1.16–3.68), BMI subgroups(< 24: RR 2.15, 95%CI 1.16–3.98; ≥ 24: RR 2.54, 95%CI 1.71–3.78), WBC subgroups(≤ 10: HR 3.12, 95%CI 1.50–6.52; > 10: RR 2.13, 95%CI 1.47–3.10), beta-block subgroups(Yes: RR 2.43, 95%CI 1.53–3.87; No: RR 1.53, 95%CI 1.05–2.22), and revascularization subgroups(Yes: RR 3.91, 95%CI 2.31–6.59; No: RR 2.17, 95%CI 1.61–2.91), and there were none significant interaction was observed.

**Table 2:** Using logistic regression to analysis Crude odds ratio and adjusted odds ratio of 28-days mortality

Variables	Crude odds ratio	*p* value	Adjusted odds ratio 95%CI	*p* value
	95%CI			
Midazolam	3.33(2.10,5.28)	0.000	2.20(1.29,3.77)	0.004
Dexmedetomidine	0.23(0.05,0.98)	0.048	0.34(0.07,1.66)	0.182
Propofol	0.34(0.22,0.54)	0.000	0.95(0.50,1.83)	0.889
MI	0.93(0.52, 1.68)	0.824		
Age	1.02(1.00,1.04)	0.021		
Hypertension	2.66(1.48,4.81)	0.001		
Heart rate	1.02(1.01,1.03)	0.020		
MBP	0.96(0.93,0.98)	0.007		
SpO2	0.81(0.73,0.90)	0.000		
WBC	1.07(1.03,1.10)	0.000		
TB	1.18(0.95,1.46)	0.129		
Glu	1.00(1.00,1.01)	0.000		
Scr	1.32(1.10,1.57)	0.002		
RASS score	1.04(0.71, 1.53)	0.842		
cTNI	1.12(1.07, 1.17)	0.000		
Aspirin	0.25(0.15,0.40)	0.000		
Betablock	0.11(0.07,0.20)	0.000	0.21(0.11,0.39)	0.000
Stain	0.22(0.13,0.35)	0.000	0.36(0.21,0.63)	0.000
Revascularization	0.25(0.15,0.40)	0.000	0.34(0.20,0.58)	0.000

Abbreviation as in Table 1

**Table 3:** Comparison of outcomes in acute myocardial farction patients between using midazolam or non-midazolam for sedative therapy

Variables	Midazolam N = 143	Non-midazolam N = 284	*p* value
Mechanical ventilation time, hours	101.6 ± 9.9	43.1 ± 4.4	0.000
CCU time, days	9.9 ± 0.9	5.6 ± 0.4	0.000
CCU mortality, n(%)	43(30.1)	36(12.7)	0.000
Hospital mortality, n(%)	47(32.9)	39(13.7)	0.000
28-days mortality, n(%)	56(39.2)	46(16.2)	0.000

*CCU* Coronary Heart Disease Intensive Care unit

### PSM

**Fig. 3:**
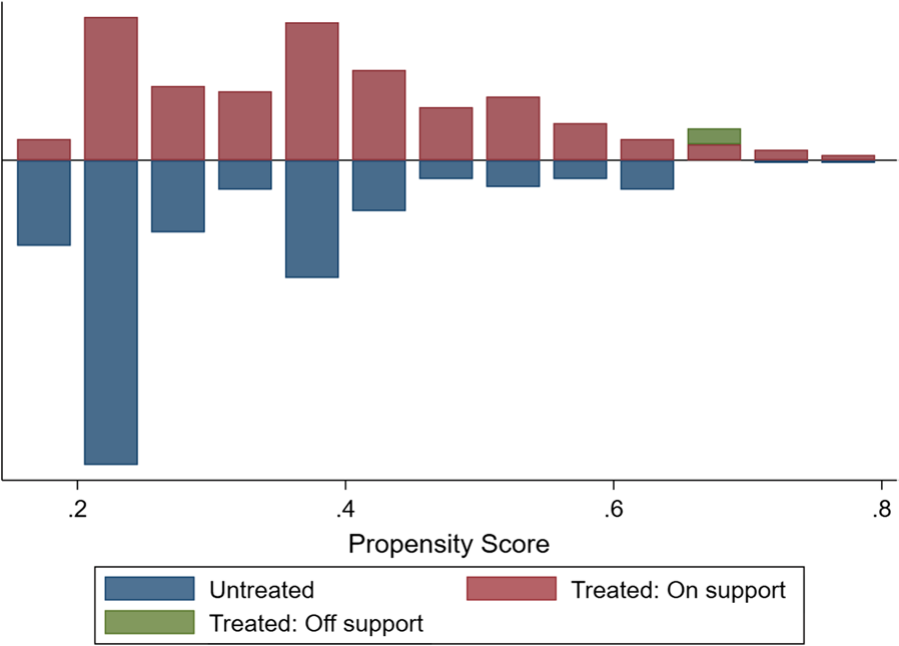
Matching graph of the propensity score before and after propensity score matching

Using PSM, 140 patients from midazolam groups and 192 from non-midazolam groups matched from each group were generated (Table 4). In order to assess the quality, we compared the standardized difference of the means and the ratio of the variances between pairs, and drawed the propensity scores (Fig. 3). None significant difference was founded between the two matched groups concerning all nine covariates. After PSM, we found that the rate of CCU mortality & hospital mortality & 28-days mortality and the length of CCU stay & mechanical ventilation were significantly higher or longer in the modazolam using.

**Table 4:** Comparison of the covariates after propensity score matching

Variables	Midazolam n = 140	Non-midazolam n = 192	*p* value
Age, years	67.0 ± 1.0	66.6 ± 0.9	0.761
Male, n(%)	96(68.5)	134(69.7)	0.182
Hypertension, n(%)	24(17.1)	25(13.0)	0.296
Scr, mg/dl	1.5 ± 0.1	1.3 ± 0.1	0.209
MI			0.578
NSTEMI, n(%)	41(29.3)	49(25.5)	
AWSTEMI, n(%)	39(27.9)	63(32.8)	
NAWSTEMI, n(%)	60(42.9)	80(41.7)	
Beta-blocker, n(%)	109(77.9)	160(83.3)	0.209
Stain, n(%)	79(56.4)	125(65.1)	0.109
Vasopressor, n(%)	113(80.7)	150(78.1)	0.566
Revascularization, n(%)	82(58.6)	129(67.2)	0.107
Clinicals outcomes			
Mechanical ventilation time, hours	103.2 ± 10.1	49.5 ± 6.0	0.000
CCU time, days	10.1 ± 0.9	6.1 ± 0.5	0.000
CCU mortality, n(%)	41(29.3)	34(17.7)	0.013
Hospital mortality, n(%)	45(32.1)	37(19.3)	0.007
28-days mortality, n(%)	54(38.6)	43(22.4)	0.001

Abbreviation as in Tables 1 and 3

### E-value

We generated an E-value to assess the sensitivity to unmeasured confounding. We determined that the observed RR of 2.41 (As shown in Fig. 2) for 28-days mortality associated with midazolam could be explained away by an unmeasured confounder that was associated with both the exposure (midazolam) and outcome (28-days mortality) by an RR of at least 4.25, above and beyond the measured confounders, but not by weaker confounding.

## Discussion

In this study, we evaluated the rate of CCU mortality, hospital mortality, 28-days mortality, and the longer of mechanical ventilation duration, CCU stay in AMI patients with sedatives therapy. Among 427 patients, the overall 28-days mortality rate was 23.9%, and mechanical ventilation using was 93.4%. Our study revealed that midazolam using for sedative therapy in AMI patients was significantly associated with longer mechanical ventilation duration and CCU stay, higher rate of CCU mortality, hospital mortality and 28-days mortality when compared to propofol or dexmedetomidine using. There was robust of result in the PSM analysis after adjustment for age, male, hypertension, Scr, MI, beta-blocker, stain, vasopressor, and revascularization. Our finding point to a negative role for midazolam in sedative therapy for AMI critical patients, which has not been reported in past study.

The primary concerns of AMI critical patients are hemodynamic and respiratory suppports. The majority of patients in our study received therapy of mechanical ventilation (93.4%) and vasopressor (78.5%). We speculated that the relatively lower rate of using aspirin (75.6%), clopidogrel (33.3%), and receiving revascularization (77%) were due to practicallly all patients undergoing mechanical ventilation and vasopressor therapy, poor physical condition, and huge risk of bleeding. Sedative therapy is necessary to increase tolerance, reduce discomfort, prevent accidental removal of instrumentation in AMI patients. In this study there were 143 patients in the midazolam using, 272 patients in propofol using, 28 patients in the dexmedetomidine using, and some of them using two or three seditives. Although propofol was reported to have vasorelaxant effect to influence myocardial perfusion and coronary flow reserve [8], due to impaired left ventricular function in AMI patients, propofol maybe result in aggressive blood pressure reduce in AMI patients. However, in our study, both propofol and dexmedetomidine using in AMI patients for sedative therapy did not show significant associated with 28-days mortality in this study. The sample size of dexmedetomidine using was relatively small in our study, and need more deeply study in future. A randomised placebo-controlled trial in past paper have showed that dexmedetomidine did not decrease postoperative atrial fibrillation in patients recovering from cardiac surgery [9].

Midazolam was showed closely associated with increased rate of 28-days mortality, and had obviously higher rate of 28-days mortality than propofol or dexmedetomidine using. This phenomenon could be attributed to the following explains. Midazolam has serious cardiorespiratory events and possible paradoxical reactions. Some cardiovascular side effects are premature ventricular contractions, vasovagal episodes, bradycardia, tachycardia, nodal rhythm, as well as variations in blood pressure and pulse rate [10]. Furthermore, midazolam have been reported as inducing coronary artery spasm [11].

We also found that when compared with propofol or dexmedetomidine, midazolam using presented with increased length of mechanical ventilation and CCU and hospital stay. Long stay in the CCU adds to the burden of health care costs. A meta-analysis demonstrated that dexmedetomidine could reduced the length of ICU stay [12]. Dexmedetomidine was founded to be similar to midazolam in terms of long-term sedation [13]. As aspect of deep sedation, midazolam significantly increased the time at target sedation [14]. It was limitation of our study that a few records of RASS scores were presented, which might attribute to the arousable and light sedation.

PSM is a powerful method to distinguish unbalanced groups. In this study, we chose age, male, hypertension, Scr, MI, beta-blocker, stain, vasopressor, and revascularization as confounding factors. And we found that compared with propofol or dexmedetomidine, midazolam using in AMI patients was still significant associated with increased rate of CCU mortality, hospital mortality, 28-days mortality, and the length of mechanical ventilation, CCU stay.

Several limitations should be reported in this study. First, potential bias remain exist as other unrecorded factors (such as the sedative and ventilation weaning protocol, pre-treatment drugs and door-to-balloon time, the incomplete records of RASS scores and serum tropoin) were not available in MIMIC III database. Instead, we performed the E-value analysis to quantify the potential implications of unmeasured confounders and found that an unmeasured confounder will not change the direction of our result. Secondly, due to the cohort design, only the association instead of causal relationship can be inferred from this study. Third, the sample size of dexmedetomidine using was relatively small, further studies are needed to explore the association between dexmedetomidine and propofol, midazolam and dexmedetomidine.

## Conclusion

Midazolam using for sedative therapy of AMI patients was significant associated with higher rate of CCU mortality, hospital mortality, 28-days mortality, and longer of mechanical ventilation duration, CCU stay, when compared to propofol or dexmedetomidine. Propofol or dexmedetomidine are preferred to be used in AMI critical patients for sedative therapy.

## Data Availability

All data are freely available with reasonable requirements from authors.

## Abbreviations

AMI: Acute myocardial infarction (AMI)
MIMIC III: Medical Information Mart for Intensive Care III
CCU: Coronary Heart Disease Intensive Care unit
BMI: Body mass index
WBC: White blood cell
PLT: Platelet
HGB: Hemoglobin
TB: Total bilirubin
Glu: Glucose
Scr: Creatinine
NSTEMI: Non-ST segment elevated myocardial infarction
AWSTEMI: Anterior wall ST segment elevated myocardial infarction
NAWSTEMI: Non-anterior wall ST segment elevated myocardial infarction
CHF: Congestive heart failure
COPD: Chronic obstructive pulmonary disease
MBP: Mean arterial pressure
